# Determinants of national health insurance enrolment among people at risk of statelessness in the Awutu Senya East Municipality and Gomoa East District of Ghana

**DOI:** 10.1186/s12913-022-08738-0

**Published:** 2023-02-14

**Authors:** Theophilus Quartey, Charles Peprah, Anthony Kwame Morgan

**Affiliations:** Department of Planning, Kwame Nkrumah University of Science and Technology, Kumasi, Ghana

**Keywords:** National Health Insurance Scheme, People at Risk of Statelessness, Determinants, Ghana, Sustainable Development Goals

## Abstract

**Background:**

This paper investigates the factors influencing the decision to enrol in Ghana’s National Health Insurance Scheme (NHIS) among people at risk of statelessness, with emphasis on the individual's demographic and socioeconomic factors.

**Methods:**

The study used data from a cross-sectional household survey undertaken in the Awutu Senya East Municipality and Gomoa East District of Ghana's Central Region between March 9 and June 26, 2021, on healthcare utilization culture among people at risk of statelessness. Descriptive statistics and binary logistic regression were used in analysing data from a sample of 384 people at risk of statelessness.

**Results:**

The results reveal that about 51% of the at-risk population have ever enrolled while 48% of the respondents were enrolled on the NHIS at the time of the survey (active members). The majority of the enrolled members acquired their membership through self-payment of the enrolment fee. Additionally, respondents aged 26–35 had higher odds of enrolling, whiles those within 56–65 years had lower odds of enrolling in health insurance. Also, persons who are married and have a high school education or an equivalent qualification were more likely to enrol, while persons with no employment were less likely to enrol.

**Conclusion:**

According to the paper, while the gap in coverage between rich and poor, married and single appears to have narrowed, these factors continue to determine NHIS coverage among people at risk of statelessness. The same is true for education. Efforts must be increased to ensure equal access to healthcare financing interventions for better access to health services.

**Supplementary Information:**

The online version contains supplementary material available at 10.1186/s12913-022-08738-0.

## Background

Globally, the realization of Universal Health Coverage (UHC) is increasingly under threat due to several issues surrounding the affordability and accessibility of healthcare services [33, 55]. The situation is worse in lower-middle-income countries (LMIC) due to unemployment [50] and high levels of poverty [5, 48]. The World Health Organisation (WHO) [67] asserts that including vulnerable populations in social health protection schemes like health insurance schemes is one of the surest ways to the realization of UHC. Countries have therefore begun to provide health insurance schemes to their citizens as a cushion to prevent the high healthcare costs that often sink many families further into poverty, the deferral of healthcare use in some instances and the resultant complications. For some who could not afford health service utilization costs, the use of herbal and faith-based healing systems became the alternative, often with catastrophic outcomes [18]. Health insurance has become a powerful tool in the achievement of the UHC [66]. The hurdle for most countries is ensuring that the population has adequate health insurance coverage. There is one category of vulnerable people that is often overlooked in (health) planning and policymaking, particularly in Africa—these are stateless populations or people at risk of statelessness [15, 62].

Statelessness denotes a situation in which a person is not regarded as national by any state under the operation of its laws [64]. People with undetermined citizenship and those with disputed citizenship risk becoming stateless. A stateless population, therefore, represents a group of people with undetermined citizenship. The condition makes it more difficult for such groups to have access to essential needs, which are regarded as basic rights. These include accessibility challenges to education, employment, and healthcare [62], demonstrating some forms of underlying vulnerabilities [15]. Globally, at least 12 million people are stateless, according to the United Nations High Commissioner for Refugees (UNHCR, even though there is no complete record of the exact number of stateless people [1, 47]. This population is estimated to be over 1 million in Africa alone, despite the absence of accurate data. Unfortunately, for Ghana and many other countries around the world, there is no data on the extent or prevalence of statelessness [15]. An earlier work by Atuguba et al. [15] has identified groups such as former refugees (from Liberia and Sierra Leone), Zongo communities, and other people who live around border towns like Aflao (close to Togo) as populations at risk of statelessness in Ghana. They are often marginalised and have to deal with problems of limited access to public services. According to UNHCR-Ghana, the possibility of becoming stateless in Ghana is not limited to in situ situations, as migrants and refugees in long-term exile in Ghana who does not have civil status documents or other individual documentation from their home country may also face statelessness [65]. With the significant marginalisation of people at risk of statelessness in terms of access to services and opportunities [62], exploring the prevalence and determinants of this groups’ enrolment in Ghana’s social health protection scheme is imperative for policy and research.

Ghana’s National Health Insurance Scheme (NHIS) was introduced in 2003 and requires every Ghanaian and foreign resident to enrol for access to basic healthcare [6, 14, 66]. The health financing scheme before the introduction of the NHIS was “cash and carry” which demanded direct payment at the point of health service use [32, 54]. Evidence exists that the “cash and carry” system, due to high healthcare costs resulted in limited access to healthcare services, especially for the poor and vulnerable populations [11, 13, 49]. The National Health Insurance Authority [NHIA], the scheme’s administrator, now sets the premium at a minimum of GH6.00 ($0.80) and a maximum of GH42.00 ($4.60), with a GH6.00 processing charge. The enrolment price for individuals under the age of 18 is GHC 6.00 ($0.80), active social security and national insurance trust [SSNIT] contributors pay GHC 6.00 ($0.80), and all other adults (18–69 years) pay GHC 28.00 ($3.73) [53]. The scheme gives premium and yearly exemptions to vulnerable groups, including children (below age 18), the elderly (70 years and above), indigents, persons with disabilities, pregnant women, and beneficiaries of the Livelihood Empowerment Against Poverty (LEAP) program. Despite these exemptions, many people have either not registered before or failed to renew their NHIS membership and continue to make out-of-pocket payments to access health services [6, 14]. For most poor and vulnerable people in Ghana who cannot afford NHIS subscriptions, family and social support [10, 12] and personal income through donations [4] have been the major source of their healthcare financing. However, according to Agyepong et al. [6], just about 40% of the entire Ghanaian population is enrolled in the scheme after more than 10 years in operation.

The literature is replete with research on the determinants (individual predictors) to enrol in health insurance [2, 14, 31, 53, 58]. In most of these studies, income and educational level, as well as sex, have been found to correlate with health insurance enrolment. Past research has always focused on either age groups-young, middle age and the elderly [22, 39, 53, 56, 66], women [27, 30, 38], pregnant women [30], rural poor [7] persons with disability [35, 63, 68] or slum dwellers [14]. In all, there is a lot of knowledge on the determinants of NHIS enrolment among a lot of vulnerable groups (the aged, women, pregnant women, slum dwellers, and people with disabilities, among others).

Despite so much focus on various vulnerable groups and geographical areas, no known study has explored the prevalence and associated factors of NHIS enrolment among people at risk of statelessness in Ghana-a notable group with much vulnerability. This is a tacit recognition that there is a lack of attention in policy discourse in health service research concerning the health and health services utilization of stateless people [40, 61]. This does not augur well for inclusivity if the UHC goal is to be achieved. Furthermore, despite the unavailability of data, pockets of evidence support the increasing population of people at risk of statelessness [1, 8, 15, 47], with significant barriers to health interventions [61]. At an age where inclusivity is vigorously championed, particularly in light of promoting the health and well-being of all [52, 68], any initiative and intervention aimed at helping reduce healthcare access and utilization inequality must be supported. The goal of this study is to fill this lacuna, by creating a new wave of research into healthcare financing for people at risk of statelessness and thereby set a tone for policy reforms that promote the welfare of stateless populations. In this paper, we investigate the factors that influence the decision to enrol in Ghana’s NHIS among people at risk of statelessness, with emphasis on the individual's demographic and socioeconomic factors. The study’s findings are envisioned to help guide policy and practice, advance the literature on health insurance enrolment, and add to the ongoing research on statelessness, health and wellbeing.

## Methods

### Study setting and design

**Fig. 1:**
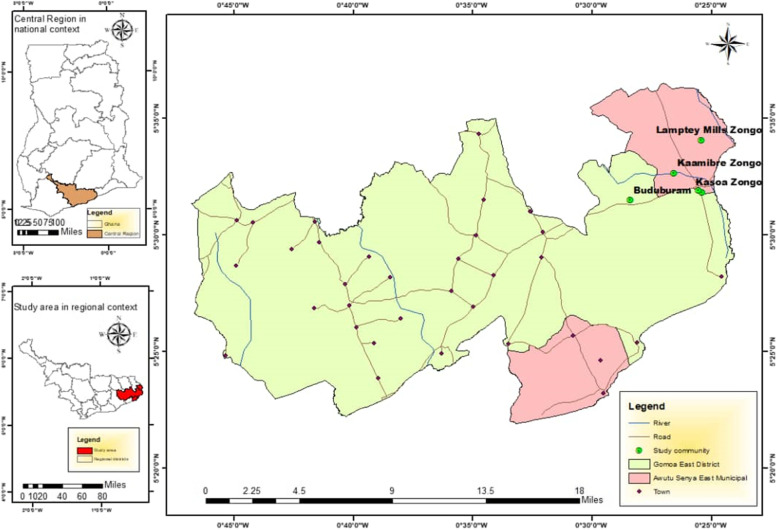
Map of the study communities. Source: Authors Construct

The study was conducted in the Central Region of Ghana. The region was selected due to the existence of many people with a higher predisposition to statelessness. The region is home to several Zongo communities and one of the largest refugee camps (the Buduburam Refugee Camp) [3]. The Awutu Senya East Municipality and the Gomoa East District were selected as the study districts (see Fig. 1 for details). The Awutu Senya East Municipal and the Gomoa East District are two of the 22 Districts in the Central Region of Ghana. The population of Gomoa East District, according to the 2010 Population and Housing Census, was 207,071 representing 9.4% of the region's total population. The Awutu Senya East Municipality has a total population of 131,543 (projected with a 2.8% growth rate) which represent 4.9% of the central region’s population according to the 2010 Population and Housing Census. Buduburam in the Gomoa East District and Kasoa Zongo, Kaamibre Zongo, Lamptey Mills Zongo, and Walantu in the Awutu Senya East Municipality were the communities purposively selected – due to the perceived prevalence of people at risk of statelessness in these communities [15].

### Research design

The study adopted the quantitative cross-sectional research design to investigate NHIS enrolment among people at risk of statelessness. Empirical researchers have occasionally utilized cross-sectional designs to describe a population of interest. We used quantitative cross-sectional data to compare subgroups within a population or to draw statistical conclusions about the population of interest (people at risk of statelessness). Cross-sectional designs are proven to help establish associations between exposure and outcome variables [28]. The research design helped in establishing the associations between the various socio-economic, and demographic variables that are associated with enrolment in the NHIS.

### Study variables

This study has both dependent and independent variables. The enrolment in the National Health Insurance Scheme is the dependent variable. This was measured as a dichotomous variable (either “Yes “or “No”). The demographic and socio-economic variables were the independent variables. The sociodemographic variables were measured as follows: sex (male or female), age (1 = below 25 years, 2 = 26–35, 3 = 36–45, 4 = 46–56, 5 = 56–65, 6 = 66 and above), marital status (1 = single, 2 = married, 3 = divorced, 4 = widowed), religion (1 = Christianity, 2 = Islam, 3 = African Traditional), educational attainment (1 = no formal education, 2 = basic education, 3 = high school, 4 = tertiary), employment status (1 = Employed, 2 = not employed).

### Sampling and recruitment procedure

The study adopted the purposive and snowballing sample techniques in selecting the participant of the study. Using [46] sample estimating formulae, a total sample size of 426 was estimated (11% non-response rate inclusive). Of the 426 questionnaires distributed, 400 were returned, of which 384 were fully completed and fit for purpose (representing a response rate of 90%). As a non-probability sampling, purposive sampling has a significant cost and time-saving benefit. It is very simple to use and suitable when probability sampling is not possible (for instance, when there is an unknown population to work with). With the purposive sampling technique, only people who have in-depth knowledge about the issue under investigation [21, 28] – enrolment in NHIS by people at risk of statelessness, were recruited. With regards to the snowball sampling technique, it enabled the researchers to depend on a limited group of known persons (people at risk of statelessness) while expanding the sample by asking the initial respondents to recommend others who should participate in the study, based on homogeneity. To circumvent the oversampling of a network of peers, exponential discriminative snowball sampling was adopted. By this, respondents provided multiple referrals, nonetheless, only one new subject was recruited among them. The choice of a new respondent is premised on the aim and objectives of the study. In this study, the population at risk of statelessness in Ghana refers to any person living in Ghana for more than 5 years (as per Sect. 10 of the citizenship Acts 2000) without any form of birth registration or any form of Ghanaian citizenship documents (Ghanaian travelling passport, or National Identification Card) and does not have evidence of citizenship to another state.

### Data collection instrument and procedure

A closed-ended questionnaire was used in data collection. The questions required respondents to pick from a restricted number of alternatives—generally multiple-choice questions with a single-word answer, ‘yes’ or ‘no,’ or a rating scale (see the attached supplementary file for details regarding the questionnaire). The purpose of using this survey instrument was to boost response rates by ensuring easier and quicker responses to questions [44]. Additionally, it also helped in obtaining measurable and quantitative data. The questions span demographic, socio-economic, health behaviour, health-seeking or healthcare utilization behaviour, and barriers to formal healthcare use. For this paper, questions covering demographic and socio-economic (under which enrolment in the NHIS was captured) were the only bands of variables used. Six research assistants, in addition to the first and third authors, were involved in the data collection which lasted between March 9 and June 26, 2021. The six research assistants had prior knowledge of mixed methods data collection and were likewise taken through the questions on two separate occasions to help them familiarize themselves with the questions. Following interactions with the research assistants, a pilot study was done to determine whether the questions are appropriate for the study and to estimate the average time a questionnaire administration lasted. This provided valuable inputs in terms of question modifications, and arrangement modifications. Although originally developed in the English language, the questions were translated into Twi, Ga, and Hausa, depending on the preference of the respondents. With the data collected during the COVID-19 pandemic, the participants and the research team adhered to guidelines (physical distancing, wearing of nose masks, and frequent hand washing) from the WHO and Ghana Health Service [51].

### Data management and analysis

Following the data collection, all the data were inputted into SPSS software (version 20). The data were checked for consistency, accuracy, and completeness. Descriptive statistical methods were employed to summarize the data using percentages and frequencies. To determine the extent to which personal characteristics influence NHIS enrolment, a binary logistic regression was performed. We developed two models to establish the factors that predict enrolment in the health protection scheme within the sample. In model 1, a univariate logistic regression was performed using sex, age, marital status, religion, length of stay in the community, educational attainment and employment status as the independent variable in each analysis and NHIS enrolment as the dependent variable. In the second model, we added all the demographic and socio-economic variables (sex, age, marital status, religion, length of stay in the community, educational attainment and employment status) as the independent variables in a multivariate logistic regression to NHIS enrolment which was the outcome variable. The models were deemed significant at the 0.05 significance level.

### Ethical consideration

All procedures complied with the institutional and/or national research committee's ethical standards, as well as the 1964 Helsinki statement and its subsequent modifications or similar ethical standards. All procedures performed in this study involving human participants follow the ethical standards of the institutional and/or national research committee and with the 1964 Helsinki declaration and its later amendments or comparable ethical standards. A retrospective exemption approval was granted by the Ghana Health Service Ethics Review Committee (GHS-ERC) according to the Standard Operating Procedures 2015. Accordingly, written and verbal consent was obtained from the respondents, while their responses were anonymously reported.

## Results

### Respondents’ characteristics

Table 1 describes the characteristics of the respondents. Up to 58.3% of the respondents are females. The age distribution of the sample revealed that 39.6% of the respondents were within the age range of 26–35 years. The study further revealed that 48.4% of the sampled respondents were married. Those who profess the Islamic faith (51.8%) were the most dominant. Up to 31.8% of the respondents have a high school education or equivalent. Further, 58.1% were employed and 86.7% of the respondents have been in their respective communities for a period equal to 5 years, but less than 10 years.

**Table 1:** Background information on the respondents

Variable	Response	Frequency (384)	Percentage (%)
Sex	Male	224	58.3
	Female	160	41.7
Age	Below 25 years	84	21.9
	26–35 years	152	39.6
	36–45 years	46	12.0
	46–55 years	61	15.9
	56–65 years	30	7.8
	66 years and above	11	2.9
Marital status	Single	141	36.7
	Married	186	48.4
	Divorced	23	6.0
	Widowed	34	8.9
Religion	Christianity	146	38.0
	Islam	199	51.8
Educational attainment	None	115	29.9
	Basic education	115	29.9
	High school	122	31.8
	Tertiary	32	8.3
Employment status	Unemployed	161	41.9
	Employed	223	58.1
Length of stay in Community	< 5 years	51	13.3
	= or > 5 years < 10 years	333	86.7

### NHIS enrolment rate among the respondents

Table 2 presents the prevalence and patterns of NHIS enrolment among the respondents. The results indicate an enrolment rate of 51%, while 48% were active members of the scheme at the time the data was collected. From this, up to 3% of the respondents have not renewed their NHIS membership, a worrying trend for that matter. Further, 95.9% of the respondents paid an enrolment fee, as they did not fall into the exempt categories. The majority paid the fees themselves. See Table 2 for more details.

**Table 2:** NHIS enrolment rate among the respondents

Variable	Response	Frequency (384)	Percentage (%)
Have you ever enrolled in the NHIS?	Yes	196	51.0
	No	188	49.0
Is your NHIS card active?	Yes	94	48.0
	No	102	52.0
Was an enrolment fee or premium paid?	Yes	188	95.9
	No	8	4.1
Who paid the enrolment fee?	Children	25	13.3
	Friend	4	2.1
	Relative	25	13.3
	NGOs	16	8.5
	Self	118	62.8

### Associated factors of NHIS enrolment

Table 3 presents the associated factors of NHIS enrolment among the respondents in both univariate and multivariate logistic regressions. Results in model 1 (the univariate logistic regression) show respondents aged 26–35 years (AOR: 2.740, CI: 1.440–5.215, *p* = 0.002) and 36–45 years (AOR: 8.091, CI: 2.850–4.967, *p* = 0.000) in addition to those who profess the Islamic faith (AOR: 5.851, CI: 3.306–10.358, *p* = 0.000) were more likely to enrol in the health protection scheme than their counterparts. Additionally, unemployed respondents were (AOR: 0.233, CI: 0.707–0.982, *p* = 0.030) less likely to enrol in the scheme, compared to their counterparts who were employed. In model 2 ((the multivariate logistic regression), respondents aged 26–35 years (AOR: 2.628, CI: 1.315–5.252, *p* = 0.004) and 36–45 years (AOR: 9.267, CI: 3.069–7.981, *p* = 0.000) were more likely to enrol in the scheme while respondents aged 56–65 years (AOR: 0.219, CI: 0.049–0.969, *p* = 0.041) were significantly less likely to enrol in the scheme. Furthermore, we found that respondents who were married (AOR: 4.684, CI: 1.338–2.383, *p* = 0.000) were more likely to enrol whereas respondents who have high school education or an equivalent educational qualification (AOR: 0.371, CI: 0.180–0.766, *p* = 0.007) and are not employed (AOR: 0.188, CI: 0.281–0.737, *p* = 0.004) were less likely to enrol in the scheme.

**Table 3:** Univariate and multivariate logistic regressions on the determinants of NHIS enrolment among the respondents

	**Univariate Logistic Regression**		**Multivariate Logistic Regression**	
**Variable**	**AOR**	**95% C.I**	**AOR**	**95% C.I**
**Demographic**				
*Sex *^***a***^				
Female	0.865	(0.529–0.914)	1.031	(1.619–2.716)
*Age (years)*^b^				
26–35 years	2.740*	**(1.440–5.215)**	2.628**	**(1.315–5.252)**
36–45 years	8.091*	**(2.850–4.967)**	**9.267**	**(3.069–7.981)**
46–55 years	0.790	(1.319–1.955)	0.592	(0.229–0.534)
56–65 years	0.295	(0.073–0.991)	0.219**	**(0.049–0.969)**
66 years and above	5.912	(0.451–0.734)	1.180	(2.345–4.875)
*Religious group *^***c***^				
Muslim	5.851*	**(3.306–10.358)**	5.201	(1.342–4.109)
Traditional	2.364	(1.984–5.679)	2.645	(2.673–3.723)
*Marital status* ^**d**^				
Married	0.720	(0.375–0.681)	4.684**	**(1.338–2.383)**
Divorced	0.528	(0.173–0.617)	0.510	(0.157–0.658)
Widowed	0.800	(0.231–0.776)	0.825	(0.223–0.756)
*Length of stay in the community *^**e**^				
= or > 5 years < 10 years	1.590	(1.802–3.150)	1.333	(1.660–2.692)
**Socio-economic**				
*Education *^***f***^				
Basic School	0.109	(0.435–0.850)	0.734	(0.361–0.793)
High School	0.782	(0.087–0.345)	0.371**	**(0.180–0.766)**
Tertiary education	3.222	(2.834–3.463)	0.667	(0.216–0.652)
*Employment status *^***g***^				
Not employed	0.233*	**(0.707–0.982)**	0.188**	**(0.281–0.737)**
**Model fitting information**				
-2Log Likelihood		425.153		344.384
Hosmer–Lemeshow χ^2^ (significance)		13.163(0.037)		11.635(0.003)
Nagelkerke R^2^		0.267		0.088

Model 1 = Demographic variables; Model 2 = All variables in Model 1 plus Socio-Economic variables

*CI* Confidence Interval, *AOR* Adjusted Odd Ratio

^**a**^Male is the reference category for the sex variable

^**b**^Below 25 years is the reference category for the age variable

^**c**^Christianity is the reference category for religious variables

^**e**^** < **5 years is the reference category for the length of stay in the community variable

^**d**^Single is the reference category for the marital status variable

^**f**^No formal education is the reference category for the education variable

^**g**^Employed is the reference category employment status variable

^*^ *p ˂ 0.05 in univariate logistic regression*

^**^ *p ˂ 0.05 in multivariate logistic regression*

## Discussion

The prevalence and associated factors of NHIS enrolment were investigated among people at risk of statelessness in the Awutu Senya East Municipality and Gomoa East District of Ghana’s Central Region. The results indicate an enrolment rate of 51%, while 48% were active members of the scheme. Further, 95.9% of the respondents paid an enrolment fee. The enrolment rate of 51% far outweighs the 17.6% reported among older adults [17]. By far, the study by Wiredu, Peprah, and Agyemang-Duah [69] among persons with disabilities reported an enrolment rate of 80%, which is significantly higher than the 51% reported in this study. While both people at risk of statelessness and people with disabilities constitute vulnerable populations, the provision of exemption for the latter might be the resulting factor that influences higher enrolments. Second, the relatively youthful population of people at risk of statelessness in the study implies that they are unqualified for age exemptions while proving indigence might be difficult, if not impossible. A third factor might be the perceived low need for healthcare services among people at risk of statelessness (due to their youthfulness), thus the low incentive to enrol in the NHIS. Regardless of the circumstances surrounding the somehow low enrolment among the respondents, measures that scale up enrolment among such a vulnerable population should be addressed.

Age was identified as a determinant of enrolment among the respondents. The findings suggest that age has a significant influence on health insurance uptake, which is similar to the findings of these scholars [20, 43, 53, 60], but differs from the findings of Brugiavini and Pace [25]. According to the Grossman model [34], health may be seen as a long-term capital stock that generates an output of healthy time. Individuals are considered to inherit an initial pool of health that depreciates with age and could be replenished via investment. This made Jütting [37] argue that as people become older, their health stock depreciates at a faster rate, demonstrating the biological process of ageing,as a result, they seek to increase their investments in health, including health insurance, in an attempt to slow the pace of the depreciation. Contrary to the Grossman model [34], however, the study established that people at risk of statelessness and aged between 56 and 65 years were significantly less likely to enrol in the scheme. While preference for traditional medicine, self-medication, and other factors could be used to explain such an anomaly, lack of economic or financial resources to support health insurance investment could also be used. The economic reason could explain why people aged between 26 and 35 years and between 36 and 45 years were significantly more likely to enrol. To this end, revising the current exemption age limit to 60 years is strongly advised, since that will correspond with the retirement age and provide more cushion to a lot of vulnerable people (including retirees).

Further, the study established a link between the marital status of the persons at risk of statelessness and enrolment in the NHIS in Ghana. Specifically, married persons were found to be more likely to enrol in health insurance compared to single, persons at risk of statelessness. A strand of literature on socio-demographic determinants of enrolment in health protection schemes reveals that higher enrolment in such health protection schemes is associated with married individuals [23, 24, 29, 41]. The higher prospect of married persons enrolling in health protection schemes is premised on the need to offset household expenditure by relying on the scheme to cater for health expenditure. On the contrary, evidence also abounds that the marital status of individuals does not predict rates of enrolment in health protection schemes [9, 57]. In addition, an association was established between higher educational attainment and enrolment in the scheme. Past studies suggest higher education chronicles a greater appreciation of health insurance schemes and a higher likelihood to enrol [25, 26, 29, 36]. That notwithstanding, [38] contended higher education could weaken the need factor [through higher awareness and abstinence from risky predisposing behaviours that necessitate frequent utilisation of health services]. Finally, persons with no employment were less likely to enrol in the scheme. Poverty and financial barriers which have been copiously documented in the literature on health economics within sub-Sahara Africa could partly explain such occurrences [16, 19, 37, 42, 45, 59]. With no employment, the likelihood to afford premiums is reduced for persons at risk of statelessness, thus hindering enrolment. 

### Strengths and limitations

First, by far, this is the only known study that examined NHIS enrolment among people at risk of statelessness in Ghana. Being so, the study has broadened the horizon and frontiers of knowledge on the determinants of enrolment in health protection schemes among another group of vulnerable people [people at risk of statelessness]. This has brought people at risk of statelessness, a largely forgotten group of vulnerable people in health literature [particularly in Africa] to the limelight [40, 61]. This should birth additional research into these vulnerable groups regarding their health and health behaviour to inform policy and practice towards attaining the health goals and targets of the Sustainable Development Goals (SDGs). Second, the identification of an association between poverty and lack of access to financial resources on the one hand and enrolment in the scheme, on the other hand, shows that by and large, the pro-rich bias associated with most health protection schemes is still at play. Without finding ways to lessen this bias, those at the bottom of the socioeconomic ladder might not reap the full benefits of the scheme.

By way of limitations, the authors were confronted with a significant barrier in terms of defining people at risk of statelessness. Ghana has no known population or defined criteria for identifying stateless populations. The authors had to, rely on the evidence presented by the respondents to categorize them as stateless or not. These inclusion and exclusion criteria were based on ownership of identification documents or cards that prove citizenship. To that end, this might impact the results of similar studies using different inclusion and exclusion criteria for defining statelessness. In future, we recommend a clearer definition of who constitutes a stateless person, to provide a more standardized and uniform definition. Methodically, the use of the purposive sampling technique brings into light some form of researcher bias. The lack of randomness could impact on generality of sample selection. That notwithstanding, we believe the approach was most appropriate since no known population of people at risk of statelessness exists, from which the samples could be randomly selected. The definition of the term is ambiguous, thus, it was most appropriate for the team to scrutinize and select only individuals who were perceived to have met the inclusion criteria. To limit the tendency of conveniently available and purposively sampling people, we combined purposive sampling with the snowballing approach, to find additional respondents who the research team could obtain. We adopted the exponential discriminative snowball sampling to circumvent the oversampling of a network of peers. By this, each subject provided multiple referrals, nonetheless, only one new subject was recruited among them, with the choice of a new subject premised on the study’s aim. Finally, the study was silent on factors such as family size, peer influence, occupation in addition to health principles, and health needs which could also influence enrolment. Future studies on the subject should include these variables, for a wider and broader understanding. That said, this does not impact the results in greater magnitude, as the included factors, were the most significant, and frequently highlighted in the literature.

## Conclusion

The determinants of national health insurance enrolment among people at risk of statelessness in the Awutu Senya East Municipality and Gomoa East District of Ghana’s Central Region were investigated in this study. The findings reveal a relatively low to moderate enrolment rate among the respondents [necessitating efforts to increase enrolment rates among them]. Age, marital status, educational attainment, and employment status were the significant associated factors of enrolment among the respondents. The study contributes to the literature on determinants of enrolment in health protection schemes, while expanding knowledge on statelessness, vulnerability, and health. Towards achieving UHC and the health targets in the SDGs, barriers to enrolment in the scheme among people at risk of statelessness must be addressed. This will require lessening financial barriers to enrolment [through capturing the qualified ones under indigent and age exemption categories]. Finally, the predictors of enrolment among people at risk of statelessness should be included in NHIS policy reforms.

## Supplementary Information

**Additional file 1:** 

## Data Availability

The datasets used and/or analysed during the current study are available from the corresponding author upon reasonable request.

## Abbreviations

AOR: Adjusted Odd Ratio
GHS-ERC: Ghana Health Service Ethics Review Committee
LEAP: Livelihood Empowerment Against Poverty
LMIC: Lower-and-Middle Income Countries
NHIS: National Health Insurance Scheme
SDGs: Sustainable development Goals
UN: United Nations
UNHCR: United Nations High Commissioner for Refugees
UHC: Universal Health Coverage
WHO: World Health Organisation

## References

[1] Abu Sulaib FM (2020). Stateless ‘bidoon’in Kuwait: a crisis of political alienation. Middle East Stud.

[2] Adebayo EF, Uthman OA, Wiysonge CS, Stern EA, Lamont KT, Ataguba JE (2015). A systematic review of factors that affect uptake of community-based health insurance in low-income and middle-income countries. BMC Health Serv Res.

[3] Agblorti SK, Grant MR (2019). Conceptualising obstacles to local integration of refugees in Ghana. Refug Surv Q.

[4] Agyemang-Duah W, Owusu-Ansah JK, Peprah C (2019). Factors influencing healthcare use among poor older females under the livelihood empowerment against poverty programme in Atwima Nwabiagya District, Ghana. BMC Res Notes..

[5] Agyemang-Duah W, Peprah C, Peprah P (2019). “Let’s talk about money”: how do poor older people finance their healthcare in rural Ghana? A qualitative study. Int J Equity Health.

[6] Agyepong IA, Abankwah DNY, Abroso A, Chun C, Dodoo JNO, Lee S, Mensah SA, Musah M, Twum A, Oh J, Park J (2016). The, “Universal” in UHC and Ghana’s National Health Insurance Scheme: policy and implementation challenges and dilemmas of a lower middle-income country. BMC Health Serv Res..

[7] Alesane A, Anang BT (2018). Uptake of health insurance by the rural poor in Ghana: determinants and implications for policy. Pan Afr Med J.

[8] Alexander H. The ethics of quantifying statelessness. In: Statelessness, governance, and the problem of citizenship. Manchester University Press; 2021. p. 238–50.

[9] Amo-Adjei J, Anku PJ, Amo HF, Effah MO (2016). Perception of quality of health delivery and health insurance subscription in Ghana. BMC Health Serv Res.

[10] Anning A. The emerging problems of the aged in Ghana: Issues of housing and basic care in selected districts in Ashanti region. Unpublished thesis submitted to the Department of Architecture and Planning, Kwame Nkrumah University of Science and Technology; 2012.

[11] Arhin-Tenkorang D. Mobilizing resources for health: the case for user fees revisited. CID Working Paper Series. 2001.

[12] Arkorful G. Sources of Support and Challenges for the Elderly in Teshie Township. Doctoral dissertation, University of Ghana; 2015.

[13] Asenso-Okyere WK, Anum A, Osei-Akoto I, Adukonu A (1998). Cost recovery in Ghana: are there any changes in health care seeking behaviour?. Health Policy Plan.

[14] Atinga RA, Abiiro GA, Kuganab-Lem RB (2015). Factors influencing the decision to drop out of health insurance enrolment among urban slum dwellers in Ghana. Tropical Med Int Health.

[15] Atuguba RA, Tuokuu FX, Gbang V (2020). Statelessness in West Africa: an assessment of stateless populations and legal, policy, and administrative frameworks in Ghana. JMHS.

[16] Asante F, Aikins M (2008). Does the NHIS cover the poor?.

[17] Ayitey AM, Nketiah-Amponsah E, Barimah A (2013). Determinants of insurance enrolment among Ghanaian adults: the case of the National Health Insurance Scheme (NHIS). Econ Manag Financ Mark.

[18] Barimah KB, Akotia CS (2015). The promotion of traditional medicine as enactment of community psychology in Ghana. J Community Psychol.

[19] Basaza R, Criel B, Van der Stuyft P (2008). Community health insurance in Uganda: why does enrolment remain low? A view from beneath. Health Policy.

[20] Bhat R, Jain N. Factoring affecting the demand for health insurance in a micro insurance scheme. Working Paper No. 2006‐07‐02. Ahmedabad: Indian Institute of Management; 2006.

[21] Bhattacherjee A. Social science research: Principles, methods, and practices. 2012. http://www.scholarcommons.com usf.edu/oa textbooks/3. Accessed 15 Mar 2022.

[22] Blanchet NJ, Fink G, Osei-Akoto I (2012). The effect of Ghana’s National Health Insurance Scheme on health care utilisation. Ghana Med J.

[23] Boateng D, Awunyor-Vitor D (2013). Health insurance in Ghana: evaluation of policy holders’ perceptions and factors influencing policy renewal in the Volta region. Int J Equity Health.

[24] Bonfrer I, Breebaart L, Van de Poel E (2016). The effects of Ghana’s national health insurance scheme on maternal and infant health care utilization. PLoS ONE.

[25] Brugiavini A, Pace N. Effects of the National Health Insurance Scheme in Ghana: Contribution to the European Report on Development. Dakar Inc. 2010.

[26] Chankova S, Sulzbach S, Diop F (2008). Impact of mutual health organizations: evidence from West Africa. Health Policy Plan.

[27] Christiani Y, Byles JE, Tavener M, Dugdale P (2017). Health insurance coverage among women in Indonesia's major cities: a multilevel analysis. Health Care Women Int.

[28] Denscombe M. The Good Research Guide for Small-Scale Social Science Projects. Buckingham: Open University Press: 2010.

[29] Dixon J, Tenkorang EY, Luginaah I (2011). Ghana's National Health Insurance Scheme: helping the poor or leaving them behind?. Eviron Plann C Gov Policy.

[30] Dixon J, Luginaah I, Mkandawire P (2014). The National Health Insurance Scheme in Ghana's Upper West Region: a gendered perspective of insurance acquisition in a resource-poor setting. Soc Sci Med.

[31] Dror DM, Hossain SS, Majumdar A, Pérez Koehlmoos TL, John D, Panda PK (2016). What factors affect voluntary uptake of community-based health insurance schemes in low-and middle-income countries? A systematic review and meta-analysis. PLoS ONE.

[32] Gibson MS. More evidence on the link between bank health and investment in Japan. J Jpn Int Econ. 1997;11(3):296–310.

[33] Gilson L (1997). The lessons of user fee experience in Africa. Health Policy Plan.

[34] Grossman HI (1972). A choice-theoretic model of an income-investment accelerator. Am Econ Rev.

[35] Howard HA (2018). The Impact of the National Health Insurance Scheme on the Lives of Persons with Disabilities in Kumasi Metropolis, Ghana. Disability, CBR & Inclusive Development.

[36] Jehu-Appiah C, Aryeetey G, Spaan E, De Hoop T, Agyepong I, Baltussen R (2011). Equity aspects of the National Health Insurance Scheme in Ghana: Who is enrolling, who is not and why?. Soc Sci Med.

[37] Jütting J. Health insurance for the poor?: determinants of participation in community-based health insurance schemes in rural Senegal. OECD Development Centre: Working Paper No. 204; 2004.

[38] Kimani JK, Ettarh R, Kyobutungi C, Mberu B, Muindi K. Determinants for participation in a public health insurance program among residents of urban slums in Nairobi, Kenya: results from a cross-sectional survey. BMC Health Serv Res. 2012;12(1):1. 10.1186/1472-6963-12-66 PMC3317843 22424445

[39] Kimani JK, Ettarh R, Warren C, Bellows B (2014). Determinants of health insurance ownership among women in Kenya: evidence from the 2008–09 Kenya demographic and health survey. Int J Equity Health.

[40] Kingston LN, Cohen EF, Morley CP (2010). Debate: Limitations on universality: the" right to health" and the necessity of legal nationality. BMC Int Health Hum Rights.

[41] Kirigia JM, Sambo LG, Nganda B, Mwabu GM, Chatora R, Mwase T (2005). Determinants of health insurance ownership among South African women. BMC Health Serv Res.

[42] Kotoh AM, Van der Geest S. Why are the poor less covered in Ghana’s national health insurance? A critical analysis of policy and practice. Int J Equity Health. 2016;15(1):1–11. 10.1186/s12939-016-0320-1 PMC4766646 26911139

[43] Kronick R, Gilmer T (1999). Explaining The Decline In Health Insurance Coverage, 1979–1995: Rising health spending levels over the past two decades have created a growing pool of uninsured workers. Health Aff.

[44] Kumekpor T (2002). Research methods & techniques of social research.

[45] Kusi A, Hansen KS, Asante FA, Enemark U. Does the National Health Insurance Scheme provide financial protection to households in Ghana?. BMC Health Serv Res. 2015;15(1):1–2. 10.1186/s12913-015-0996-8 PMC4537562 26275412

[46] Lwanga SK, Lemeshow S, World Health Organization. Sample size determination in health studies: a practical manual. World Health Organization; 1991.

[47] Lysaker O. Transnational Struggle for Recognition: Axel Honneth on the Embodied Dignity of Stateless Persons. In: Migration, Recognition and Critical Theory. Cham: Springer; 2021. p. 91–115.

[48] Mahembe E, Odhiambo NM (2018). The dynamics of extreme poverty in developing countries. Studia Universitatis Vasile Goldiș Arad, Seria Științe Economice.

[49] McIntyre D, Thiede M, Dahlgren G, Whitehead M (2006). What are the economic consequences for households of illness and of paying for health care in low-and middle-income country contexts?. Soc Sci Med.

[50] Mehry EB, Ashraf S, Marwa E (2021). The impact of financial inclusion on unemployment rate in developing countries. Int J Econ Financ Issues.

[51] Morgan AK (2020). Making COVID-19 prevention etiquette of social distancing a reality for the homeless and slum dwellers in Ghana: lessons for consideration. Local Environ.

[52] Morgan AK, Ibrahim R, Awafo BA (2021). Creating an Inclusive Society: The Role of Ethnic Social Movements in Promoting Equality and Inclusion in Ghana. Ethiop J Soc Sci Vol.

[53] Morgan AK, Adei D, Agyemang-Duah W, Mensah AA (2022). An integrative review on individual determinants of enrolment in National Health Insurance Scheme among older adults in Ghana. BMC Primary Care.

[54] Nyonator F, Kutzin J (1999). Health for some? The effects of user fees in the Volta Region of Ghana. Health Policy Plan.

[55] Okungu V, Chuma J, Mulupi S, McIntyre D (2018). Extending coverage to informal sector populations in Kenya: design preferences and implications for financing policy. BMC Health Serv Res.

[56] Osei-Akoto I, Adamba C. Ethnic and religious diversity as determinants of health insurance uptake in Ghana. Fourth European Conference for African Studies. In: 4th European Conference on African Studies Uppsala. 2011.

[57] Owusu-Sekyere E, Chiaraah A (2014). Demand for Health Insurance in Ghana: what factors influence enrolment?. Am J Public Health Res.

[58] Sarker AR, Sultana M, Mahumud RA, Ahmed S, Islam Z, Morton A, Khan JA (2017). Determinants of enrollment of informal sector workers in cooperative based health scheme in Bangladesh. PLoS ONE.

[59] Sarpong N, Owusu-Dabo E, Kreuels B, Fobil JN, Segbaya S, Amoyaw F, Hahn A, Kruppa T, May J (2015). Prevalence of malaria parasitaemia in school children from two districts of Ghana earmarked for indoor residual spraying: a cross-sectional study. Malar J.

[60] Savage E, Wright D. Health insurance and health care utilization: theory and evidence from Australia 1989–90. CHERE; 2001.

[61] Suphanchaimat R, Kantamaturapoj K, Putthasri W, Prakongsai P (2015). Challenges in the provision of healthcare services for migrants: a systematic review through providers’ lens. BMC Health Serv Res.

[62] Suphanchaimat R, Kantamaturapoj K, Pudpong N, Putthasri W, Mills A (2016). Health insurance for people with citizenship problems in Thailand: a case study of policy implementation. Health Policy Plan.

[63] Szilagyi PG. Health insurance and children with disabilities. Future Child. 2012;1:123–48. 10.1353/foc.2012.0000 22550688

[64] United Nations [UN]. Statelessness & Nationality/Citizenship. Panel discussion: Preventing and Reducing Statelessness (20 May 2011), Persons at Risk of Statelessness in Serbia. 2011.

[65] UNHCR – Ghana. Study on Statelessness in Ghana. Terms of Reference. UNHCR; 2016.

[66] Van Der Wielen N, Falkingham J, Channon AA (2018). Determinants of National Health Insurance enrolment in Ghana across the life course: Are the results consistent between surveys?. Int J Equity Health..

[67] WHO. Arguing for Universal Health Coverage. 2013. https://apps.who.int/iris/handle/10665/204355. Accessed 10 Oct 2021.

[68] Wang F (2012). Measurement, optimization, and impact of health care accessibility: a methodological review. Ann Assoc Am Geogr.

[69] Wiredu DN, Peprah C, Agyemang-Duah W (2021). Prevalence of health insurance enrolment and associated factors among persons with disabilities in Ghana. Cogent Med.

