# Supplementary material for: Determinants of national health insurance enrolment among people at risk of statelessness in the Awutu Senya East Municipality and Gomoa East District of Ghana
Source: BMC Health Serv Res. 2023 Feb 14;23:153. doi: 10.1186/s12913-022-08738-0 (PMC9927045; doi:10.1186/s12913-022-08738-0)
Supplement: Supplementary file 1 — Additional file 1: [file 12913_2022_8738_MOESM1_ESM.docx]

**DEPARTMENT OF PLANNING**

**COLLEGE OF ART AND BUILT ENVIRONMENT**

**KWAME NKRUMAH UNIVERSITY OF SCIENCE AND TECHNOLOGY, KUMASI**


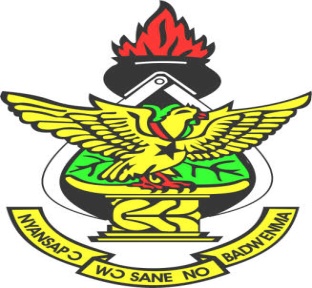


**QUESTIONNAIRE FOR THE PEOPLE AT RISK OF STATELESSNESS (UNDETERMINED CITIZENS)**

**INTRODUCTION AND INFORMED CONSENT FORM**

Dear Respondent,

I am a Master of Philosophy Student at the Department of Planning, Kwame Nkrumah University of Science and Technology (KNUST), Kumasi, Ghana. I am conducting a study on “**An Investigation into the Formal Healthcare Utilization Culture among Population at Risk of Statelessness (Undetermined Citizenship) in Ghana.”** Your district and community have been chosen as study regions. I wish that you take full participation in the study through interviews/questionnaire administration. The data you provide will be used in a research activity intended to produce scientific knowledge. It will also help in designing health policy that addresses the health care needs of the population at risk of statelessness (undetermined citizenship) in Ghana as a whole. Nonetheless, participation in this study is completely voluntary and you reserve the right to decide not to respond to certain questions or withdraw at any time in the course of the interview/ questionnaire administration without any penalty. By signing or thumb printing this form, you are giving your consent to participate in the study. Notice that whatever data you disclose will only be used for academic purposes and will be treated as strictly confidential as possible and that will be reported in a way that no one will know your specific responses. If you have any questions and queries regarding this study, please do not hesitate to contact me at 0244637349/0205819773. You may also contact my Supervisor, Dr. Charles Peprah (0200198245) from Department of Planning KNUST, Kumasi.

Thank you for your participation.

Sincerely,

**SIGNED**

**THEOPHILUS QUARTEY**

DEPARTMENT OF PLANNING

KNUST, Kumasi.

**-------------------------------------------------------------------------------------------------**

**Signature/ Thumb Print of Respondent**

**SECTION A: DEMOGRAPHIC CHARACTERISTICS**

| **S/N** | **QUESTION** | **RESPONSE** | **CODE** | **SKIP** |
| --- | --- | --- | --- | --- |
| **101** | Gender | Male  Female | 1 [ ]  2 [ ] |  |
| **102** | Age | Below 25 years  26-35  36-45  46-55  56-65  66 and above | 1 [ ]  2 [ ]  3 [ ]  4 [ ]  5 [ ]  6 [ ] |  |
| **103** | Were you born in Ghana? | Yes  No | 1 [ ]  2 [ ] |  |
| **104** | Is any of your parents Ghanaian? | Yes  No | 1 [ ]  2 [ ] | **If No, Skip to 106** |
| **105** | If yes, which of them? | Mother  Father | 1 [ ]  2 [ ] |  |
| **106** | Ethnic Group | Akan  Northerner  Ewe  Others | 1 [ ]  2 [ ]  3 [ ]  4 [ ] |  |
| **107** | Religion | Christianity  Islam  African Traditional Religion  Others | 1 [ ]  2 [ ]  3 [ ]  4 [ ] |  |
| **108** | Marital Status | Single  Married  Divorced  Widowed | 1 [ ]  2 [ ]  3 [ ]  4 [ ] |  |
| **109** | How long have you lived in Ghana? | <7 years  ≥7 years | 1 [ ]  2 [ ] |  |
| **110** | Do you possess any Ghanaian Identification Documents(GID)? | Yes  No | 1 [ ]  2 [ ] |  |
| **111** | Which of the following GID do you possess? Tick as apply. | Birth Certificate  Travelling Passport  Voters ID card  NHIS Card  Ghana Card | 1 [ ]  2 [ ]  3 [ ]  4 [ ]  5 [ ] |  |
| **112** | Length of stay in the community? | <5 years  ≥5 years | 1 [ ]  2 [ ] |  |
| **113** | Are you living with your spouse? | Yes  No | 1 [ ]  2 [ ] |  |
| **114** | If no, who do you stay with? | Alone  Family  Children  Others | 1 [ ]  2 [ ]  3 [ ]  4 [ ] |  |
| **115** | Which region do you live? | Volta Region  Central Region  Greater Accra Region  Ashanti Region | 1 [ ]  2 [ ]  3 [ ]  4 [ ] |  |

**SECTION B: SOCIO-ECONOMIC CHARACTERISTICS**

| **S/N** | **QUESTION** | **RESPONSE** | **CODE** | **SKIP** |
| --- | --- | --- | --- | --- |
| **201** | Education | No formal education  Basic school education  High school education  College/tertiary | 1 [ ]  2 [ ]  3 [ ]  4 [ ] |  |
| **202** | Employment status | Not employed  Employed | 1 [ ]  2 [ ] | If Not employed, Skip to **204** |
| **203** | Occupation | Farming  Artisanal work  Industrial work  Civil public service  Others | 1 [ ]  2 [ ]  3 [ ]  4 [ ]  5 [ ] |  |
| **204** | Monthly income (GH¢) | Below 500.00  501.00-700.00  701.00-900.00  901.00-1000.00  1001.00 and above | 1 [ ]  2 [ ]  3 [ ]  4 [ ]  5 [ ] |  |
| **205** | Household size | 1-3  4-6  7-10  ≥11 | 1 [ ]  2 [ ]  3 [ ]  4 [ ] |  |
| **205** | How many children do you have? | None  1  2  3  4  5 and above | 1 [ ]  2 [ ]  3 [ ]  4 [ ]  5 [ ]  6 [ ] | **If None, Skip to 208** |
| **206** | How many of your children are female? | None  1  2  3  4  5 and above | 1 [ ]  2 [ ]  3 [ ]  4 [ ]  5 [ ]  6 [ ] |  |
| **207** | How many of your children are male? | None  1  2  3  4  5 and above | 0 [ ]  1 [ ]  2 [ ]  3 [ ]  4 [ ]  5 [ ] |  |
| **208** | Have you ever registered for health insurance? (NHIS) | Yes  No | 1 [ ]  2 [ ] | **If No, Skip to 214** |
| **209** | Is your NHIS active? | Yes  No | 1 [ ]  2 [ ] | **If yes, Skip to 211** |
| **210** | If no, why is your NHIS inactive? | Do not know it must be renewed annually  Have not been sick  Enrollment fee/premium is too expensive  Travel time is too high  Travel cost is too high  Waiting time at the renewal site is too long  Poor quality care for those paying with NHIS  Preferred services not covered  Use clinics/traditional healers that don’t accept NHIS | 1 [ ]  2 [ ]  3 [ ]  4 [ ]  5 [ ]  6 [ ]  7 [ ]  8 [ ]  9 [ ] |  |
| **211** | Was a fee/premium paid for when last renewed | Yes  No | 1 [ ]  2 [ ] |  |
| **212** | Who paid for the fee? | Children  LEAP  Friend  Relative  NGO  Exempt/did not pay  Self | 1 [ ]  2 [ ]  3 [ ]  4 [ ]  5 [ ]  6 [ ]  7 [ ] |  |
| **213** | Are you covered by any type of supplementary health insurance? | Yes  No | 1 [ ]  2 [ ] |  |
| **214** | Do you engage in any indoor recreation activities? | Yes  No | 1 [ ]  2 [ ] |  |
| **215** | Do you engage in any outdoor recreation activities? | Yes  No | 1 [ ]  2 [ ] |  |
| **216** | Do you have any remittance from family members? | Yes  No | 1 [ ]  2 [ ] |  |
| **217** | Would you say you are economically active? | Yes  No | 1 [ ]  2 [ ] |  |
| **218** | Can you read and write in any language? | Yes  No | 1 [ ]  2 [ ] |  |
| **219** | Do you have any autonomy in determining the kind of health care you seek for? | Yes  No | 1 [ ]  2 [ ] |  |

**SECTION C: HEALTH STATUS VARIABLES**

| **S/N** | **QUESTIONS** | **RESPONSE** | **CODE** | **SKIP** |
| --- | --- | --- | --- | --- |
| **301** | Have you been ill in the past 3 months? | Yes  No | 1 [ ]  2 [ ] |  |
| **302** | In general, how would you rate your health today? | Very good  Good  Fair  Poor  Very poor | 1 [ ]  2 [ ]  3 [ ]  4 [ ]  5 [ ] |  |
| **303** | Good health (very good, good, fair), and poor health(poor and very poor) | Good Health  Poor Health | 1 [ ]  2 [ ] |  |
| **304** | Compared to one year ago, how would you rate your health in general now? | Much better now  Somewhat better now  About the same  Somewhat worse now  Much worse now | 1 [ ]  2 [ ]  3 [ ]  4 [ ]  5 [ ] |  |
| **305** | Do you have any communicable disease? | Yes  No | 1 [ ]  2 [ ] | **If No, Skip to 307** |
| **306** | If yes, which of the communicable disease do you suffer from?  (Tick all that apply) | Malaria  Tuberculosis  Hepatitis B  Cholera  Diarrhea  Skin diseases  Others specify | 1 [ ]  2 [ ]  3 [ ]  4 [ ]  5 [ ]  6 [ ]  7 [ ] |  |
| **307** | Have you been diagnosed of any chronic Non- Communicable Diseases? | Yes  No | 1 [ ]  2 [ ] | **If No, Skip to 309** |
| **308** | Which of the chronic illnesses were you diagnosed of?  (Tick all that apply) | Diabetes  Hypertension  High blood pressure  Asthma  Respiratory disease  Cancers  Stroke  Chronic kidney disease  Depression  Arthritis  Insomnia  Mental disorder  Disorder of joint and bones  Eye problem  Ear problem | 1 [ ]  2 [ ]  3 [ ]  4 [ ]  5 [ ]  6 [ ]  7 [ ]  8 [ ]  9 [ ]  10 [ ]  11 [ ]  12 [ ]  13 [ ]  14 [ ]  15 [ ] |  |
| **309** | Do you suffer from bodily pains? | Yes  No | 1 [ ]  2 [ ] | **If No, Skip to 311** |
| **310** | Do you take medicine for pains? | Yes  No | 1 [ ]  2 [ ] |  |
| **311** | During the past 12 months, how many times were you seriously injured? | None  1  2  3  4  5 | 1 [ ]  2 [ ]  3 [ ]  4 [ ]  5 [ ]  6 [ ] |  |
| **312** | Do you go for monthly health checkups? | Yes  No | 1 [ ]  2 [ ] |  |
| **313** | Have you ever had a hearing test? | Yes  No | 1 [ ]  2 [ ] | If **No, Skip to 315** |
| **314** | If yes, do you currently use hearing aid? | Yes  No | 1 [ ]  2 [ ] |  |
| **315** | Have you ever had an eye test? | Yes  No | 1 [ ]  2 [ ] | If **No, Skip to 316** |
| **316** | If yes, do you currently use a glass? | Yes  No | 1 [ ]  2 [ ] |  |
| **317** | Do you have any disability? | Yes  No | 1 [ ]  2 [ ] | If **No, Skip to 319** |
| **318** | If yes, which of the following disabilities do you have? | Hearing  Visual  Locomotion  Speech | 1 [ ]  2 [ ]  3 [ ]  4 [ ] |  |
| **319** | How would you compare your health to the rest of the people within your locality? | Better than them  Same as them  Worse than them | 1 [ ]  2 [ ]  3 [ ] |  |

**SECTION D: HEALTH BEHAVIOUR VARIABLES**

| **PHYSICAL ACTIVITY** | | | | |
| --- | --- | --- | --- | --- |
| **S/N** | **QUESTIONS** | **RESPONSE** | **CODE** | **SKIP** |
| **401** | How physically active are you compared to other aged? | Less active  Same  More active | 1 [ ]  2 [ ]  3 [ ] |  |
| **402** | For the past one month, have you undertaken any physical activity? | Yes  No | 1 [ ]  2 [ ] | If **No, Skip to 407** |
| **403** | How many days in the last week did you walk for at least 30 minutes in total? | One day  Two days  Three days  Four days  Five days  More days | 1 [ ]  2 [ ]  3 [ ]  4 [ ]  5 [ ]  6 [ ] |  |
| **404** | How many days in the last week did you do moderate activities such as dancing etc. for at least 30minutes in total? | One day  Two days  Three days  Four days  Five days  More days | 1 [ ]  2 [ ]  3 [ ]  4 [ ]  5 [ ]  6 [ ] |  |
| **405** | How many days in the last week did you do vigorous activities such as running, gardening/yard work etc. for at least 30minutes in total? | One day  Two days  Three days  Four days  Five days  More days | 1 [ ]  2 [ ]  3 [ ]  4 [ ]  5 [ ]  6 [ ] |  |
| **406** | Are they any reasons that keep you from being more physically active? | Yes  No | 1 [ ]  2 [ ] |  |
| **ALCOHOL USE** | | | | |
| **407** | Have you ever consumed alcohol before? | Yes  No | 1 [ ]  2 [ ] | **If No, Skip to 413** |
| **408** | In the past one year, have you ever consumed alcohol? | Yes  No | 1 [ ]  2 [ ] | **If No, Skip to 413** |
| **409** | How often did you consume alcohol in the last one year? | Every day  Every week  Every two weeks  Every month  Every three months  More than three months | 1 [ ]  2 [ ]  3 [ ]  4 [ ]  5 [ ]  6 [ ] |  |
| **410** | How many times have you consumed alcohol in the past one year? | 1  2  3  4  5 or more | 1 [ ]  2 [ ]  3 [ ]  4 [ ]  5 [ ] |  |
|  |  |  |  |  |
| **411** | Which of the following drinks did you consume? (tick all that apply) | Beer  Guinness  Pito  Palm wine  Akpteshi  Wine  Spirits | 1 [ ]  2 [ ]  3 [ ]  4 [ ]  5 [ ]  6 [ ]  7 [ ] |  |
| **412** | Which type of alcohol? | Foreign  Local | 1 [ ]  2 [ ] |  |
| **TOBACCO USE** | | | | |
| **413** | Have you ever smoked tobacco or smokeless tobacco before? | Yes  No | 1 [ ]  2 [ ] | If **no, Skip to 419** |
| **414** | In the past one year, have you smoked before? | Yes  No | 1 [ ]  2 [ ] | If **No, Skip to 420** |
| **415** | If yes, how many times have you smoked in the past one year? | 1  2  3  4  5 or more | 1 [ ]  2 [ ]  3 [ ]  4 [ ]  5 [ ] |  |
| **416** | How frequent do you smoke? | Every day  Every week  Every two weeks  Every month  Every three months  More than three months | 1 [ ]  2 [ ]  3 [ ]  4 [ ]  5 [ ]  6 [ ] |  |
| **417** | On the average, how many cigarettes do you usually smoke each week? | Less than 20  More than 20 | 1 [ ]  2 [ ] |  |
| **418** | Which of the following tobacco products do you smoke? (Tick all that apply). | Tawa  Cigarettes  Cigars  Chewing tobacco  Snuff powder | 1 [ ]  2 [ ]  3 [ ]  4 [ ]  5 [ ] |  |
| **419** | Is there anybody in your family who smokes? | Yes  No | 1 [ ]  2 [ ] |  |
| **FRUITS INTAKE** | | | | |
| **420** | In the last one month, have you eaten fruits? | Yes  No | 1 [ ]  2 [ ] | If **No, Skip to 424** |
| **421** | In the last one month, how many times per week did you usually eat fruits? | 1 time per week  2  3  4  5 or more time per week | 1 [ ]  2 [ ]  3 [ ]  4 [ ]  5 [ ] |  |
| **422** | How frequent do you eat fruits? | Every day  2-5 days  Every week  Every two weeks  Every three weeks  Every month | 1 [ ]  2 [ ]  3 [ ]  4 [ ]  5 [ ]  6 [ ] |  |
| **423** | Which of the following fruits did you consume (tick all that apply)? | Oranges  Pineapple  Watermelon  Banana  Guava  Pear  Mango  Pawpaw  Others specify | 1 [ ]  2 [ ]  3 [ ]  4 [ ]  5 [ ]  6 [ ]  7 [ ]  8 [ ]  9 [ ] |  |
| **VEGETABLES INTAKE** | | | | |
| **424** | In the last one month, have you eaten vegetables? | Yes  No | 1 [ ]  2 [ ] | If **No, Skip to 501** |
| **425** | During the past one month, how many times per week did you usually eat vegetables? | 1 time per week  2  3  4  5 or more time per week | 1 [ ]  2 [ ]  3 [ ]  4 [ ]  5 [ ] |  |
| **426** | How frequent do you eat vegetables? | Every day  2-5 days  Every week  Every two weeks  Every three weeks  Every month | 1 [ ]  2 [ ]  3 [ ]  4 [ ]  5 [ ]  6 [ ] |  |
| **427** | Which of the vegetables did you consume? (Tick all that apply). | Kontomire  Garden eggs  Cabbage  Okro  Tomatoes  Carrot  Ayoyo  Others specify | 1 [ ]  2 [ ]  3 [ ]  4 [ ]  5 [ ]  6 [ ]  7 [ ]  8 [ ] |  |

**SECTION E: PREVALENCE AND PATTERNS OF HEALTH CARE USE**

| **S/N** | **QUESTIONS** | **RESPONSE** | **CODE** | **SKIP** |
| --- | --- | --- | --- | --- |
| **501** | Have you ever sought formal health care for your health problems in the last one year? | Yes  No | 1 [ ]  2 [ ] | **If No, Skip** to **504** |
| **502** | How many times have you sought formal health care in the last one year? | 1  2  3  4  5 or more | 1 [ ]  2 [ ]  3 [ ]  4 [ ]  5 [ ] |  |
| **503** | How often did you seek formal health? | Every week  Every two weeks  Every month  Every three months  More than three months | 1 [ ]  2 [ ]  3 [ ]  4 [ ]  5 [ ] |  |
| **504** | Have you used informal health care services in the last one year? | Yes  No | 1 [ ]  2 [ ] | **If No, Skip to 507** |
| **505** | How many times did you seek informal healthcare in the last one year? | 1  2  3  4  5 or more | 1 [ ]  2 [ ]  3 [ ]  4 [ ]  5 [ ] |  |
| **506** | How often did you seek informal health care in the last one year? | Every week  Every two weeks  Every month  Every three months  More than three months | 1 [ ]  2 [ ]  3 [ ]  4 [ ]  5 [ ] |  |
| **507** | Have you sought health care (both formal and informal) in the last one year? | Yes  No | 1 [ ]  2 [ ] | **If at least one of these questions; Q501 & 504 is No, Tick No for Q507, and Skip to Q510 if Q501 is Yes** |
| **508** | How often did you seek health care (both formal and informal) in the last one year? | Every week  Every two weeks  Every month  Every three months  More than three months | 1 [ ]  2 [ ]  3 [ ]  4 [ ]  5 [ ] |  |
| **509** | How many times did you seek for health care services (that is a hybrid of formal and informal health care)? | 1  2  3  4  5 or more | 1 [ ]  2 [ ]  3 [ ]  4 [ ]  5 [ ] |  |
| **510** | What type of health provider or facility did you consult most? | Public health facility  Private health facility | 1 [ ]  2 [ ] |  |
| **511** | What kind of health facility (either public or private) do you normally use? | Hospital  Clinic  Health centre  CHPS compound | 1 [ ]  2 [ ]  3 [ ]  4 [ ] |  |
| **512** | How early did you seek health care for your health problems after detecting the symptoms? | Immediately/in a day  2-3 days  4-6 days  Between a week and on month  More than a month | 1 [ ]  2 [ ]  3 [ ]  4 [ ]  5 [ ] |  |
| **513** | Who mostly decide the type of health care for you? | Yourself  Family members  Friends  Health care providers | 1 [ ]  2 [ ]  3 [ ]  4 [ ] |  |
| **514** | Where do you normally seek health care information from? | Health professionals  Family  Friends  Media  Drug stores  Literature | 1 [ ]  2 [ ]  3 [ ]  4 [ ]  5 [ ]  6 [ ] |  |
| **515** | How many kilometers do you cover to access/use healthcare? | ≥1km  2km  3km  4km  5 or more | 1 [ ]  2 [ ]  3 [ ]  4 [ ]  5 [ ] |  |
| **516** | How do you get to the health facility? | Walking  Motor bike  Bicycle  Commercial vehicle  Private vehicle  Tricycle/ Aboboyaa | 1 [ ]  2 [ ]  3 [ ]  4 [ ]  5 [ ]  6 [ ] |  |
| **517** | How long do you wait at the health facility before you see a doctor? | Less than 20minutes  20-40 minutes  More than 40 minutes | 1 [ ]  2 [ ]  3 [ ] |  |
| **518** | How much do you spend when you visit health care? | Less than GH ¢100  GH¢ 100- GH¢200  More than GH¢200 | 1 [ ]  2 [ ]  3 [ ] |  |
| **519** | Which of the following best describes your regular source of fund to pay for your health care cost? | Personal income  NHIS subscription  Social support network  Family members  Friends  LEAP | 1 [ ]  2 [ ]  3 [ ]  4 [ ]  5 [ ]  6 [ ] |  |
| **520** | Which health care item do you mostly spend your income on? | Health aide  Long-term care  Ambulance  In-patient  Out-patient  Drugs | 1 [ ]  2 [ ]  3 [ ]  4 [ ]  5 [ ]  6 [ ] |  |
| **521** | How would you assess the level of treatment you received from the health care providers? | Very good  Good  Fair  Poor  Very poor | 1 [ ]  2 [ ]  3 [ ]  4 [ ]  5 [ ] |  |
| **522** | How would you rate the quality of services offered by the health care providers? | Very good  Good  Fair  Poor  Very poor | 1 [ ]  2 [ ]  3 [ ]  4 [ ]  5 [ ] |  |
| **523** | How would you rank the attitude of the health care providers? | Very good  Good  Fair  Poor  Very poor | 1 [ ]  2 [ ]  3 [ ]  4 [ ]  5 [ ] |  |
| **524** | In general, are you satisfied with health care use? | Yes  No | 1 [ ]  2 [ ] |  |

**SECTION F: BARRIERS**

| **S/N** | **QUESTIONS** | **RESPONSE** | **CODE** | **SKIP** |
| --- | --- | --- | --- | --- |
| **601** | Do you face any barrier(s) in your quest to use health care? | Yes  No | 1 [ ]  2 [ ] | **If No, End the survey** |
| **602** | Which of the following barriers do you normally encounter? | Transportation problem  Distance (long travel time)  Financial problem/income  Poor attitude of healthcare providers  Language problem  Cultural beliefs  Long waiting time  High healthcare cost/expenditure  Lack of knowledge on health care  Others specify | 1 [ ]  2[ ]  3 [ ]  4 [ ]  5 [ ]  6 [ ]  7 [ ]  8 [ ]  9 [ ]  10 a[ ] |  |
| **604** | To what extent do the barriers interfere your health care use? | To a large extent  To a moderate extent  To a smaller extent | 1 [ ]  2 [ ]  3 [ ] |  |
| **605** | Have you ever delayed in seeking or stopped seeking healthcare due to lack of money? | Yes  No | 1 [ ]  2 [ ] |  |

##

## APPENDIX TWO

**INTERVIEW GUIDE - POPULATION AT RISK OF STATELESSNESS**

**SECTION A: EXTENT OF HEALTH CARE USE**

1. Have you used formal health care in the last 12 months?
2. If yes, why do you use formal health care?
3. How frequent do you seek formal health care?
4. Have you used informal health care in the last 12 months?
5. If yes, why do you use informal health care?
6. How frequent do you use informal health care?
7. Which health type (formal or informal) do you normally use most and why?
8. Do you use private health facility? If yes, why?
9. Do you use public health facility and why?
10. Which health facility (private or public) do you normally use most and why?
11. Why do you use either in-patient or outpatient services?
12. How early or late do you seek formal health care?
13. What are the main sources of funding for your health care?
14. Which health items do you mostly spend your income on and why?
15. What kind of health information do you seek and from whom?

**SECTION B: BARRIERS OF HEALTH CARE USE**

1. What are the main barriers you encounter in your quest to use health care?
2. How have these barriers interfered with your use of health care?
3. In general, kindly describe to me your experience in visiting health care facility?
4. In general, what would you suggest must be done to improve health care use?
